# Mechanisms regulating spill‐over of synaptic glutamate to extrasynaptic NMDA receptors in mouse substantia nigra dopaminergic neurons

**DOI:** 10.1111/ejn.13075

**Published:** 2015-10-20

**Authors:** A. R. Wild, M. Bollands, P. G. Morris, S. Jones

**Affiliations:** ^1^ Department of Physiology, Development & NeuroscienceUniversity of CambridgeDowning StreetCambridgeCB2 3DYUK; ^2^ Present address: Department of PharmacologyUniversity of Colorado School of MedicineAuroraCO80045USA

**Keywords:** glutamate transporter, metabotropic glutamate receptor, NMDA glutamate receptor, substantia nigra pars compacta

## Abstract

*N*‐Methyl‐d‐aspartate glutamate receptors (NMDARs) contribute to neural development, plasticity and survival, but they are also linked with neurodegeneration. NMDARs at synapses are activated by coincident glutamate release and depolarization. NMDARs distal to synapses can sometimes be recruited by ‘spill‐over’ of glutamate during high‐frequency synaptic stimulation or when glutamate uptake is compromised, and this influences the shape of NMDAR‐mediated postsynaptic responses. In substantia nigra dopamine neurons, activation of NMDARs beyond the synapse during different frequencies of presynaptic stimulation has not been explored, even though excitatory afferents from the subthalamic nucleus show a range of firing frequencies, and these frequencies change in human and experimental Parkinson's disease. This study reports that high‐frequency stimulation (80 Hz/200 ms) evoked NMDAR‐excitatory postsynaptic currents (EPSCs) that were larger and longer lasting than those evoked by single stimuli at low frequency (0.1 Hz). MK‐801, which irreversibly blocked NMDAR‐EPSCs activated during 0.1‐Hz stimulation, left a proportion of NMDAR‐EPSCs that could be activated by 80‐Hz stimulation and that may represent activity of NMDARs distal to synapses. TBOA, which blocks glutamate transporters, significantly increased NMDAR‐EPSCs in response to 80‐Hz stimulation, particularly when metabotropic glutamate receptors (mGluRs) were also blocked, indicating that recruitment of NMDARs distal to synapses is regulated by glutamate transporters and mGluRs. These regulatory mechanisms may be essential in the substantia nigra for restricting glutamate diffusion from synaptic sites and keeping NMDAR‐EPSCs in dopamine neurons relatively small and fast. Failure of glutamate transporters may contribute to the declining health of dopamine neurons during pathological conditions.

## Introduction

*N*‐Methyl‐d‐aspartate glutamate receptors (NMDARs) have critical functional roles in the mammalian CNS, where they influence neural development, plasticity and survival. However, under certain conditions NMDARs can also trigger excitotoxic cell death (Hardingham & Bading, 2010; Paoletti *et al*., 2013; Wyllie *et al*., 2013). These outcomes are influenced by the shape of NMDAR‐excitatory postsynaptic currents (EPSCs) and the associated calcium influx, but the regulation of NMDAR‐EPSC size and duration in substantia nigra pars compacta (SNc) dopamine neurons is relatively unknown (Wild *et al*., 2014). This is of potential importance because NMDARs contribute to burst firing and synaptic plasticity in midbrain dopamine neurons (Bonci & Malenka, 1999; Blythe *et al*., 2007; Harnett *et al*., 2009). Furthermore, NMDAR‐mediated excitotoxicity is a putative contributing factor to the progressive degeneration of SNc dopamine neurons in Parkinson's disease (PD; Ambrosi *et al*., 2014), and NMDAR antagonists are considered potential therapies in the face of limited treatment options for patients with PD (Little & Brown, 2014).

NMDAR‐EPSC shape is determined primarily by the concentration of glutamate in the synaptic cleft and the properties of synaptic NMDARs (Clements, 1996; Bergles *et al*., 1999). At some synapses, high‐frequency synaptic stimulation causes released glutamate to ‘spill‐over’ from the synaptic cleft (Rosenmund *et al*., 1995; Asztely *et al*., 1997; Clark & Cull‐Candy, 2002; Harris & Pettit, 2007, 2008) and activate extrasynaptic NMDARs, defined as NMDARs located more than 100 nm from the synapse (Papouin & Oliet, 2014). Extrasynaptic NMDARs can also be recruited during low‐frequency stimulation when glutamate uptake is compromised, and by tonic glutamate release (Sah *et al*., 1989; Chen & Diamond, 2002; Clark & Cull‐Candy, 2002; Angulo *et al*., 2004; Fellin *et al*., 2004; Le Meur *et al*., 2007; Milnerwood *et al*., 2010). Extrasynaptic NMDARs can influence the shape of the NMDAR‐EPSC, and either extrasynaptic NMDARs alone (Hardingham & Bading, 2010) or co‐activation of synaptic and extrasynaptic NMDARs (Zhou *et al*., 2013) can trigger cell death. The contributions of ‘spill‐over’ and ambient glutamate to NMDAR activity in SNc dopamine neurons have not been determined.

SNc dopamine neurons lack dendritic spines for excitatory synaptic transmission (Tepper *et al*., 1987). It was hypothesized that glutamate might readily diffuse to and activate NMDARs distal to the synapse (putative extrasynaptic NMDARs) during high‐frequency presynaptic stimulation. In mouse SNc dopamine neurons, the proportion of NMDARs activated by 80‐Hz stimulation was regulated by glutamate transporters (that limit diffusion via binding and removal of extracellular glutamate), particularly when metabotropic glutamate receptors (mGluRs) were also blocked. This mechanism may be essential for restricting glutamate to synaptic sites and shaping the NMDAR‐EPSC in SNc dopamine neurons. The failure of glutamate transporters may contribute to the declining health of dopamine neurons when pathological conditions challenge the synapse, such as increased high‐frequency activity of excitatory afferents from the subthalamic nucleus, which occurs in the SNc of human patients with PD and in animal models of PD (Magnin *et al*., 2000; Piallat *et al*., 2011).

## Materials and methods

### Slice preparation

Male C57Bl6 mice aged 17–25 postnatal days were decapitated under isoflurane anaesthesia in accordance with the Animals (Scientific Procedures) Act UK (1986) and Local Ethical Committee approval. The brain was removed into ice‐cold slicing solution composed of (in mm): NaCl, 75; sucrose, 100; glucose, 25; NaHCO_3_, 25; KCl, 2.5; CaCl_2_, 1; MgCl_2_, 4; NaH_2_PO_4_, 1.25; kynurenic acid, 0.25, maintained at pH 7.4 by bubbling with 95% O_2_ and 5% CO_2_. Horizontal midbrain slices (250 μm) containing the substantia nigra were prepared using a Campden 7000smz Vibrating Microtome (Campden Instruments, UK). Slices were transferred to a submersion incubation chamber containing a modified recording solution of composition (in mm): NaCl, 125; glucose, 25; KCl, 2.5; NaHCO_3_, 26; NaH_2_PO_4_, 1.26, MgCl_2_, 4; CaCl_2_, 1, bubbled with 95% O_2_ and 5% CO_2_ and maintained at 30 °C for 1–6 h prior to use. Slices were transferred to the stage of an Olympus BX51W upright microscope, and SNc dopamine neurons were viewed at a magnification of × 600 using differential interference contrast optics. The chamber was perfused at 2–3 mL/min with oxygenated recording solution at 30 ± 2 °C (as above but with 10 mm glucose, 0.1 mm MgCl_2_). Patch pipettes were pulled from thin‐walled borosilicate glass (GC150F, Harvard Apparatus, Kent, UK) to low resistances [2–3 MΩ when filled with pipette solution containing (in mm): CsCH_3_SO_3_, 130; CsCl, 5; NaCl, 2.8; 4‐(2‐hydroxyethyl)‐1‐piperazineethanesulphonic acid (HEPES), 20; ethylene glycol tetraacetic acid (EGTA), 5; CaCl_2_, 0.5; MgCl_2_, 3; Mg‐ATP, 2; Na‐GTP, 0.3; pH ~7.2], in order to keep the series resistance as low as possible and minimize errors arising from poor voltage clamp control of distal dendrites of neurons in brain slices.

### Electrophysiology

A total of 92 dopamine neurons from 92 slices (prepared from 58 mice) were used for data recordings for this study. Neurons were voltage‐clamped to −50 mV using an Axopatch 200B patch‐clamp amplifier (Axon Instruments, USA), and the membrane current was low‐pass‐filtered at 2 kHz then sampled at 20 kHz using a Micro 1401 controlled by Spike 2 (Version 4) software (Cambridge Electronic Design, Cambridge, UK). Series resistance (typically 4–6 MΩ) was compensated by up to 40%. Synaptic currents were evoked using a bipolar stainless‐steel electrode (Frederick Haer, USA) placed rostral to the recorded cell at an approximate distance of 0.5 mm; stimuli (200 μs duration; stimulation intensity, 50–150 μA) were applied at frequencies specified in the text in the presence of low extracellular Mg^2+^ (0.1 mm), 6,7‐dinitroquinoxaline‐2,3‐dione (DNQX; 10 μm), picrotoxin (50 μm) and glycine (10 μm; all from Sigma‐Aldrich UK) to isolate NMDAR responses.

SNc dopamine neurons were identified by their anatomical location and the presence of a prominent inward current (*I*_sag_) during a voltage step from −60 to −110 mV. This current is representative of *I*_h_, a time‐dependent inward current mediated by hyperpolarization‐activated cyclic nucleotide‐gated (HCN) channels, which is routinely used to identify SNc dopamine neurons (Washio *et al*., 1999; Neuhoff *et al*., 2002; Margolis *et al*., 2006; Ungless & Grace, 2012). Between 70% and 90% of SNc neurons are thought to be dopaminergic (Fallon & Loughlin, 1995; Nair‐Roberts *et al*., 2008).

### Data analysis

NMDAR‐EPSC amplitudes (peak minus baseline current) in response to low‐frequency stimulation (0.1 Hz) were measured in Spike 2 from the average of 10 NMDAR‐EPSCs in control solution and during or following drug treatment. NMDAR‐EPSC amplitudes (peak minus baseline current) in response to high‐frequency stimulation (80 Hz/200 ms) were measured in Spike 2 from three control responses prior to and one‐three responses during or following drug treatment. All currents recorded at −50 mV were inward, but for clarity they are plotted in graphs as positive amplitudes. The total charge transfer during NMDAR‐EPSCs in response to different frequencies of synaptic stimulation was calculated from averaged responses as the integral of the current using:

Q=∑(I·t)

where *Q* is the total charge transfer (pC), *I* is the current amplitude (pA) of each data point sampled in the EPSC, and *t* is the time between sampling (50 μs for a sampling frequency of 20 kHz).

The time constant of the decay of NMDAR‐EPSCs in response to low‐ or high‐frequency stimuli was calculated from a fit of a two‐exponential function (Stocca & Vicini, 1998; Vicini *et al*., 1998; Brothwell *et al*., 2008) to the peak‐to‐baseline decay of an average of 10 EPSCs for 0.1 Hz or three EPSCs for 80 Hz (using WinWCP version 4.7.4 software, available at http://spider.science.strath.ac.uk/sipbs/software_ses.htm):

$$I(t)=A_{1}e^{-\frac{t}{\tau_{1}}}+A_{2}e^{-\frac{t}{\tau_{2}}}$$

The weighted time constant (τ_w_) was calculated as:

$$\tau_{w}=\tau_{1}\left(\frac{A_{1}}{A_{1}+A_{2}}\right)+\tau_{2}\left(\frac{A_{1}}{A_{1}+A_{2}}\right)$$

### Statistics

Data are expressed as mean ± standard error; the *n* values refer to the number of cells. To test whether data sets showed a normal distribution, the Shapiro–Wilk normality test was used. For statistical comparisons, the significance level was set to 0.05. The Student's two tailed *t*‐test was used to compare two normally distributed groups of data, non‐parametric tests (as reported in the text) were used for two groups that were not both normally distributed, and for three or more groups of data, one‐way anova with Tukey *post hoc* tests (or non‐parametric tests) were used. Statistical analysis was performed using GraphPad Prism 4 (version 4.01), GraphPad Software (La Jolla, CA, USA).

### Materials

All drugs, including d‐2‐amino‐5‐phosphonopentanoate (D‐AP5; 50 μm), D,L‐AP5 (100 μm), dizocilpine (MK‐801; 20 μm), memantine (10 μm; Sigma‐Aldrich UK), the glutamate transporter inhibitor d,l‐threo‐benzyloxyaspartic acid (TBOA; 30 μm), the Group II mGluR antagonist LY 341495 (200 nm) and tetrodotoxin (TTX; 100 nm; all Tocris UK) were added to the perfusion solution.

## Results

### NMDAR‐EPSCs in response to low‐ vs. high‐frequency synaptic stimulation

**Figure 1:**
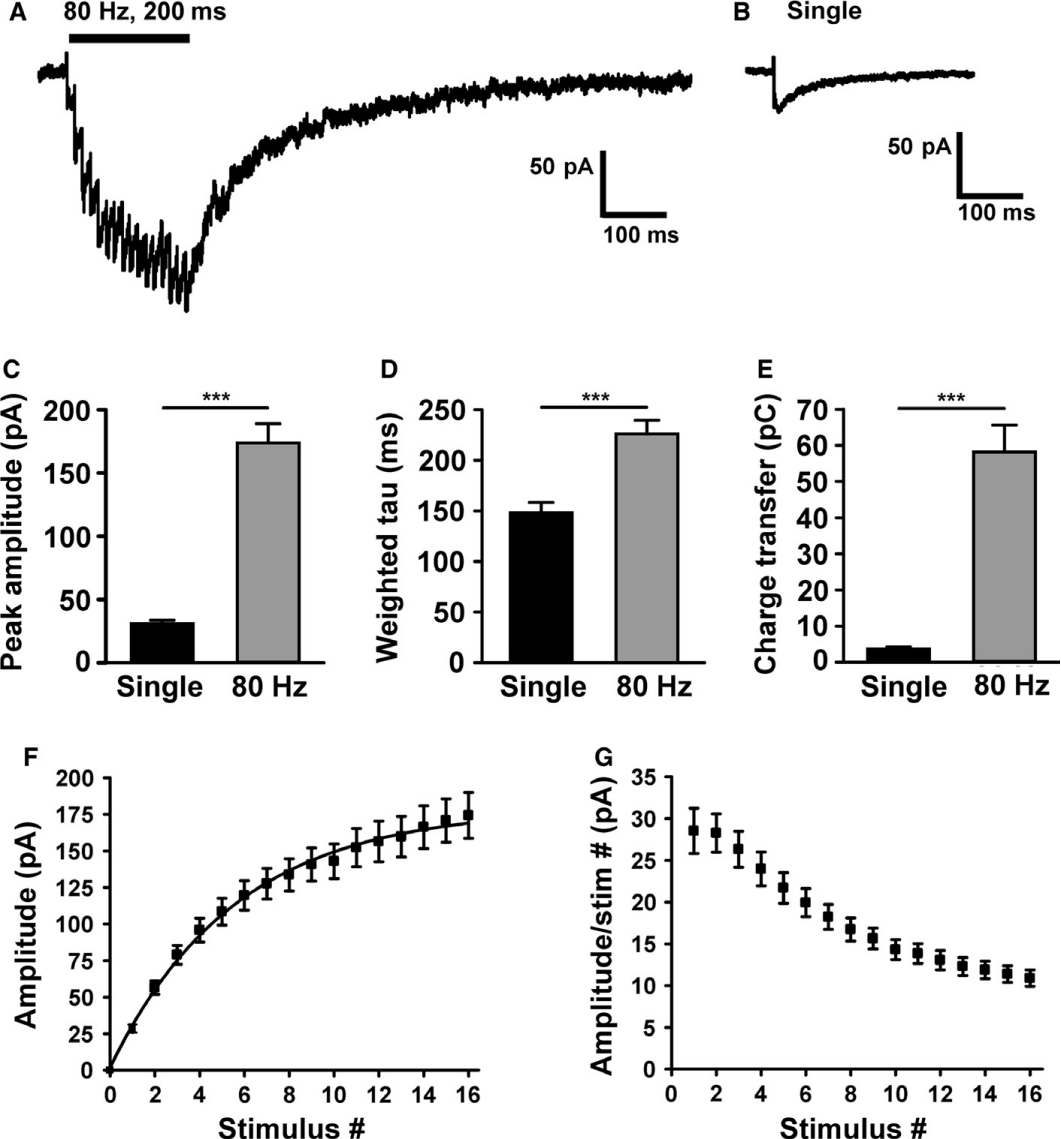
*N*‐methyl‐d‐aspartate glutamate receptor (NMDAR) responses in substantia nigra pars compacta (SNc) dopamine neurons to high‐ and low‐frequency presynaptic stimulation. (A) Example recording from a SNc dopamine neuron of a NMDAR‐excitatory postsynaptic current (EPSC) in response to high‐frequency presynaptic stimulation (80 Hz for 200 ms; average of three responses). This represents current through synaptic and possibly extrasynaptic NMDARs. (B) Example recording from the same SNc dopamine neuron of a NMDAR‐EPSC in response to a single presynaptic stimulus (average of 10 responses delivered at 0.1 Hz). Quantification of (C) the peak amplitude, (D) the decay time constant and (E) the transfer of charge in response to single stimuli vs. 80‐Hz stimuli (*n *= 27; ***P < 0.0001). (F) Cumulative amplitude of NMDAR responses to each of the 16 stimuli in the 80 Hz burst (*n *= 27; peak amplitude after 16th stimulus is significantly greater than peak amplitude after 1st stimulus, *P *< 0.0001). (G) Peak amplitude during the NMDAR response to 80‐Hz stimulation, divided by the stimulus number (*n *= 27; peak amplitude in response to the 16th stimulus is significantly smaller than peak amplitude in response to the 1st stimulus, *P *< 0.0001).

Figure 1 shows NMDAR‐EPSCs in SNc dopamine neurons in response to high‐frequency stimulation (80 Hz for 200 ms; Fig. 1A) and low‐frequency stimulation (0.1 Hz; Fig. 1B). NMDAR‐EPSCs in response to 80‐Hz stimulation were significantly larger in amplitude (*P *< 0.0001, paired *t*‐test), longer in decay (*P *< 0.0001, Wilcoxon matched pairs test) and greater in charge transfer (*P *< 0.0001, Wilcoxon matched pairs test; *n *= 27; Fig. 1C–E). D,L‐AP5 (100 μm) inhibited the responses to 0.1‐Hz and 80‐Hz stimulation by 91.3 ± 2% and 93 ± 1.4%, respectively (*n *= 5; not shown). The amplitude of NMDAR‐EPSCs in response to each consecutive stimulus during 80‐Hz stimulation declined, as the response amplitude to the last stimulus was significantly less than the response amplitude to the first stimulus (*P *< 0.0001, paired *t*‐test; Fig. 1F and G). This may reflect depletion of the releasable pool of vesicles (Rizzoli & Betz, 2005) or changes in NMDAR properties, or both, but conventional facilitation was not apparent. Thus, no evidence was seen that an increase in glutamate release might account for the increased NMDAR‐EPSC in response to 80 Hz compared with single stimuli.

### High‐frequency stimulation recruits extrasynaptic NMDARs

**Figure 2:**
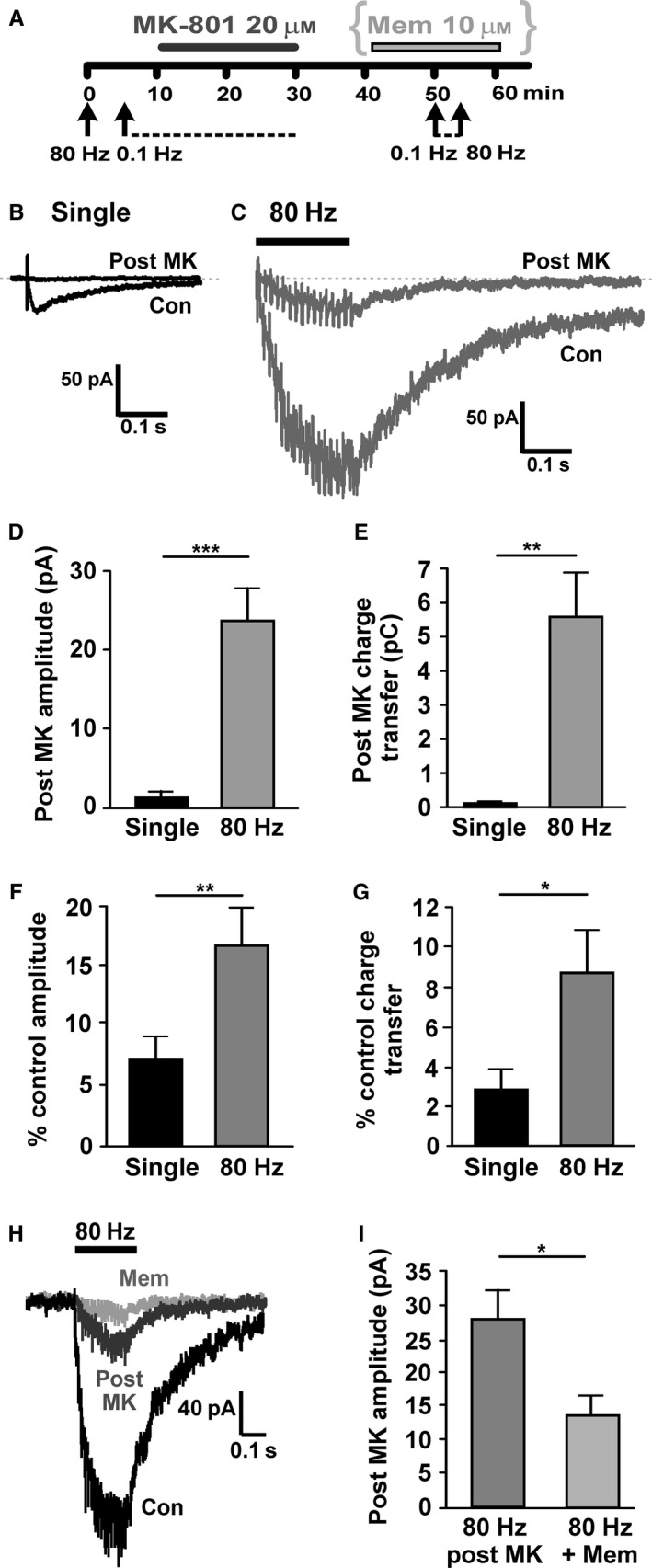
Isolation of extrasynaptic *N*‐methyl‐d‐aspartate glutamate receptors (NMDARs) in substantia nigra pars compacta (SNc) dopamine neurons. (A) Experimental protocol for determining MK‐801 block of single and 80‐Hz‐evoked NMDAR‐excitatory postsynaptic currents (EPSCs) and (in H, I) the effect of memantine after MK‐801 block. (B) Example recording from a SNc dopamine neuron of NMDAR‐EPSCs in response to single presynaptic stimuli (average of six–10 responses delivered at 0.1 Hz) before (con) and after (post MK) perfusion with MK‐801 (20 μm for 20 min, combined with 0.1‐Hz stimulation of synaptic NMDARs). In theory, this represents the inhibition of synaptic NMDARs. (C) Example recording from the same SNc dopamine neuron of a NMDAR‐EPSC in response to high‐frequency presynaptic stimulation (80 Hz for 200 ms; average of three responses) before (con) and after (post MK) perfusion with MK‐801 (as in A). In theory, the latter represents the removal of synaptic NMDAR component in response to 80 Hz, leaving only the extrasynaptic NMDAR component. (D) Quantification of the peak amplitude of NMDAR responses to 0.1‐Hz and 80‐Hz stimuli that remains after MK‐801 block of synaptic current; significantly more current remains for 80‐Hz stimulation (***P = 0.0003). (E) Quantification of the transfer of charge in response to 0.1‐Hz and 80‐Hz stimuli that remains after MK‐801 block of synaptic current; significantly more charge transfer remains for 80‐Hz stimulation (**P = 0.005). (F) The peak amplitude of the current remaining after MK‐801 block is expressed as a percentage of control amplitude; significantly greater percentage remains for 80‐Hz stimulation (**P = 0.003). (G) Charge transfer remaining after MK‐801 block is expressed as a percentage of control charge transfer; significantly greater percentage remains for 80‐Hz stimulation (*P = 0.02). (D–G) *n *= 8–9. (H) Example recording from a SNc dopamine neuron of a NMDAR‐EPSC in response to high‐frequency presynaptic stimulation (80 Hz for 200 ms) before (con) and after (post MK) perfusion with MK‐801 and then after (mem) perfusion with memantine. (I) Quantification of the peak amplitude of NMDAR responses to 80‐Hz stimulation that remain after MK‐801 block of synaptic current, and then following perfusion with memantine (10 μm for 20 min); significantly less current remains following memantine inhibition (*P = 0.03; *n *= 6).

Glutamate released by 80‐Hz stimulation may evade binding and removal by glutamate transporters, enabling it to ‘spill‐over’ from the synapse and activate more distal NMDARs, so‐called extrasynaptic NMDARs, effectively increasing the number of NMDARs contributing to the EPSC. This was explored by applying the use‐dependent and irreversible NMDAR antagonist, MK‐801 (20 μm; Rosenmund *et al*., 1995). Synaptic NMDARs have been defined as those activated by single synaptic stimuli (Harris & Pettit, 2007); if MK‐801 is applied during low‐frequency synaptic stimulation, when synaptic NMDAR channels are opened (Fig. 1B), it should irreversibly block synaptic NMDARs while leaving any inactive extrasynaptic NMDARs unblocked (Rosenmund *et al*., 1995). Prior to MK‐801 application, control NMDAR‐EPSCs were evoked by 0.1‐Hz and 80‐Hz stimuli (Fig. 2A–C). MK‐801 was then applied for 20 min during 0.1‐Hz stimulation, followed by 20 min of washing out MK‐801 in the absence of stimulation (Fig. 2A) to remove unbound MK‐801 and prevent block of subsequent 80 Hz‐evoked NMDAR‐EPSCs. This protocol led to near‐complete and irreversible block of NMDAR‐EPSCs in response to 0.1‐Hz stimulation (Fig. 2B), reducing the amplitude (from −29.6 ± 5.2 pA to −1.8 ± 0.4 pA, *n *= 9; *P *= 0.0007, paired *t*‐test) and charge transfer (from 4.05 ± 0.89 pC to 0.15 ± 0.07 pC, *n *= 8; *P *= 0.004, paired *t*‐test). Following a 20‐min wash in MK‐801‐free solution, 80‐Hz stimulation was delivered. The resulting NMDAR‐EPSC was significantly inhibited when compared with control responses prior to MK‐801 applicat‐ion (Fig. 2C), with significant changes in amplitude (from −186.2 ± 35.3 pA to −23.40 ± 4.0 pA, *n *= 9; *P *= 0.001, paired *t*‐test) and charge transfer (from 80.76 ± 19.41 pC to 5.59 ±1.3 pC, *n *= 8; *P *= 0.005, paired *t*‐test). Nonetheless, 80‐Hz stimulation evoked NMDAR‐EPSCs that were significantly larger in amplitude (*P *= 0.0003, paired *t*‐test; Fig. 2D) and charge transfer (*P *= 0.005, paired *t*‐test; Fig. 2E) than NMDAR‐EPSCs in response to 0.1‐Hz stimulation, indicating that a population of NMDARs could be recruited by 80‐Hz stimulation after block of synaptic NMDARs. The percentage of the 80 Hz‐evoked NMDAR‐EPSC after MK‐801 block was 16.3 ± 3.3% of control amplitude (Fig. 2F) and 8.4 ± 2.0% of control charge transfer (Fig. 2G), significantly more than the percentage amplitude and charge transfer of the remaining 0.1 Hz‐evoked NMDAR‐EPSC (*P *= 0.003 and *P *= 0.02, respectively), suggesting that ~8–16% of the 80‐Hz‐evoked NMDAR‐EPSC is due to extrasynaptic NMDARs.

It has previously been shown in rat SNc dopamine neurons that NMDARs activated during 80‐Hz stimulation are substantially inhibited by memantine, while responses to 0.1‐Hz stimulation are not (Wild *et al*., 2013; see also Wu & Johnson, 2015). To determine whether memantine would block the extrasynaptic component of the 80 Hz‐evoked NMDAR‐EPSC in mouse SNc dopamine neurons, memantine (10 μm) was applied following MK‐801 block of synaptic NMDARs (Fig. 2A). The 80 Hz‐evoked NMDAR‐EPSC remaining after MK‐801 block (28 ± 4.4 pA) was significantly reduced by memantine (to 13.5 ± 3 pA, *n *= 6; Fig. 2H; *P *= 0.03, Wilcoxon matched pairs test).

In control experiments where MK‐801 was not applied, the amplitudes of responses to either 0.1‐Hz or 80‐Hz stimuli were not significantly different over a similar time‐course (not shown, *n *= 4 in each case, *P *= 1.13 and *P *= 0.63, respectively, Wilcoxon matched pairs tests), indicating that the observed reductions in NMDAR‐EPSCs were not due to rundown. In three of the experiments shown in Fig. 2D, D‐AP5 (50 μm) was added after MK‐801 block; the remaining current measured in response to 80‐Hz stimulation in the presence of D‐AP5 was 4.1 ± 0.4 pA (not shown, *n *= 3).

### Ambient glutamate causes tonic activation of NMDARs

**Figure 3:**
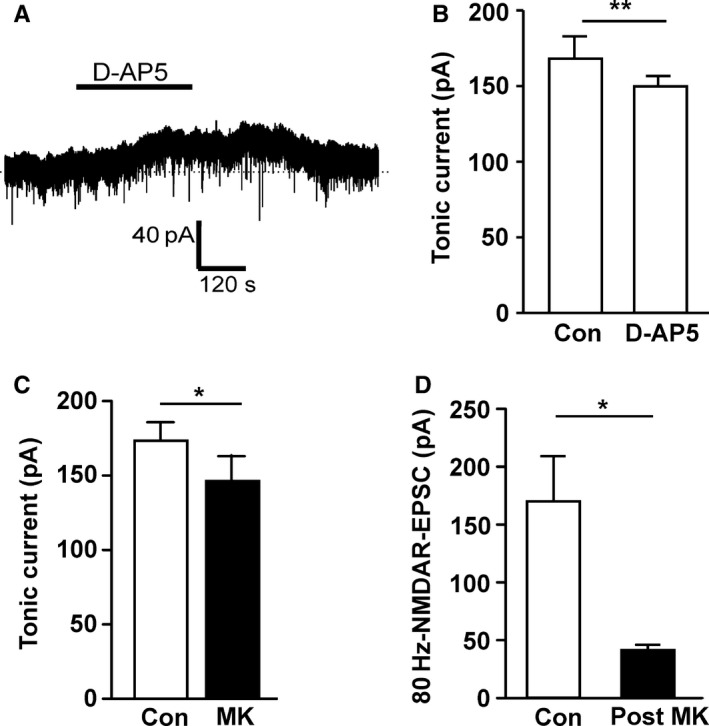
Ambient glutamate causes tonic activation of *N*‐methyl‐d‐aspartate glutamate receptors (NMDARs). (A) Example recording from a substantia nigra pars compacta (SNc) dopamine neuron of tonic (holding) current at −50 mV (in control conditions: 0.1 mm Mg^2+^, 50 μm picrotoxin, 10 μm glycine and 10 μm DNQX) before, during and after perfusion of D‐AP5 (50 μm), which caused a reversible reduction in inward current. (B) Quantification of tonic current at −50 mV in control conditions and in D‐AP5 (*n *= 8; **P = 0.001). (C) Quantification of the effect of MK‐801 (20 μm) on tonic current amplitude (*n *= 12; *P = 0.02). (D) Following perfusion with MK‐801, as in Fig. 2 but without 0.1‐Hz stimulation, a significant reduction in the NMDAR response to 80‐Hz stimulation was still observed (*n *= 6; *P = 0.03).

Next it was considered whether the population of extrasynaptic NMDARs in dopamine neurons was in fact active during MK‐801 application and therefore available for use‐dependent block, causing underestimation of the relative size of this population of NMDARs. This could be explained if ambient levels of glutamate are present in the SNc, causing tonic activation of NMDARs. Tonic NMDAR current activated by ambient extracellular glutamate has been reported in hippocampal slice preparations (Sah *et al*., 1989; Angulo *et al*., 2004; Fellin *et al*., 2004; Le Meur *et al*., 2007) but has not been reported in the SNc. To test for tonic NMDAR activity in dopamine neurons, the holding current at −50 mV was recorded and the NMDAR competitive antagonist, D‐AP5 (50 μm), was applied. This caused a significant reduction of inward holding current of 17.3 ± 3.2 pA (*n *= 8; *P *= 0.001, paired *t*‐test; Fig. 3A and B). In the presence of TTX (100 nm) to block action potential‐dependent glutamate release, D‐AP5 also caused a significant reduction of inward current (11.9 ± 3.4 pA, *n *= 7; *P *= 0.01, paired *t*‐test) that was not significantly different to the effect of D‐AP5 in control conditions (*P *= 0.27, unpaired *t*‐test). This suggests that a tonic NMDAR current due to ambient glutamate exists in brain slices containing SNc. This might have enabled MK‐801 to block tonically active extrasynaptic NMDARs. In support of this, MK‐801 also caused a significant reduction in inward holding current of 27.0 ± 0.3 pA (*n *= 12; *P *= 0.01, paired *t*‐test; Fig. 3C).

In light of this, we tested whether MK‐801 would block 80 Hz‐evoked NMDAR‐EPSCs if it was applied and washed out for the same durations as shown in Fig. 2A but in the absence of 0.1‐Hz synaptic stimulation during the application. It was found that 80 Hz‐evoked NMDAR‐EPSCs were significantly reduced by MK‐801 application, to 26.4 ± 6% of the control amplitude (*n *= 6; *P *= 0.03, Wilcoxon matched pairs test; Fig. 3D), indicating that MK‐801 block does not require stimulated glutamate release. It therefore appears likely that ambient levels of glutamate cause NMDAR channels to open, allowing MK‐801 block, and that this may have caused the underestimation of the component of 80 Hz‐evoked NMDAR‐EPSCs that is extrasynaptic.

### Mechanisms regulating glutamate spill‐over from excitatory synapses on SNc dopamine neurons

**Figure 4:**
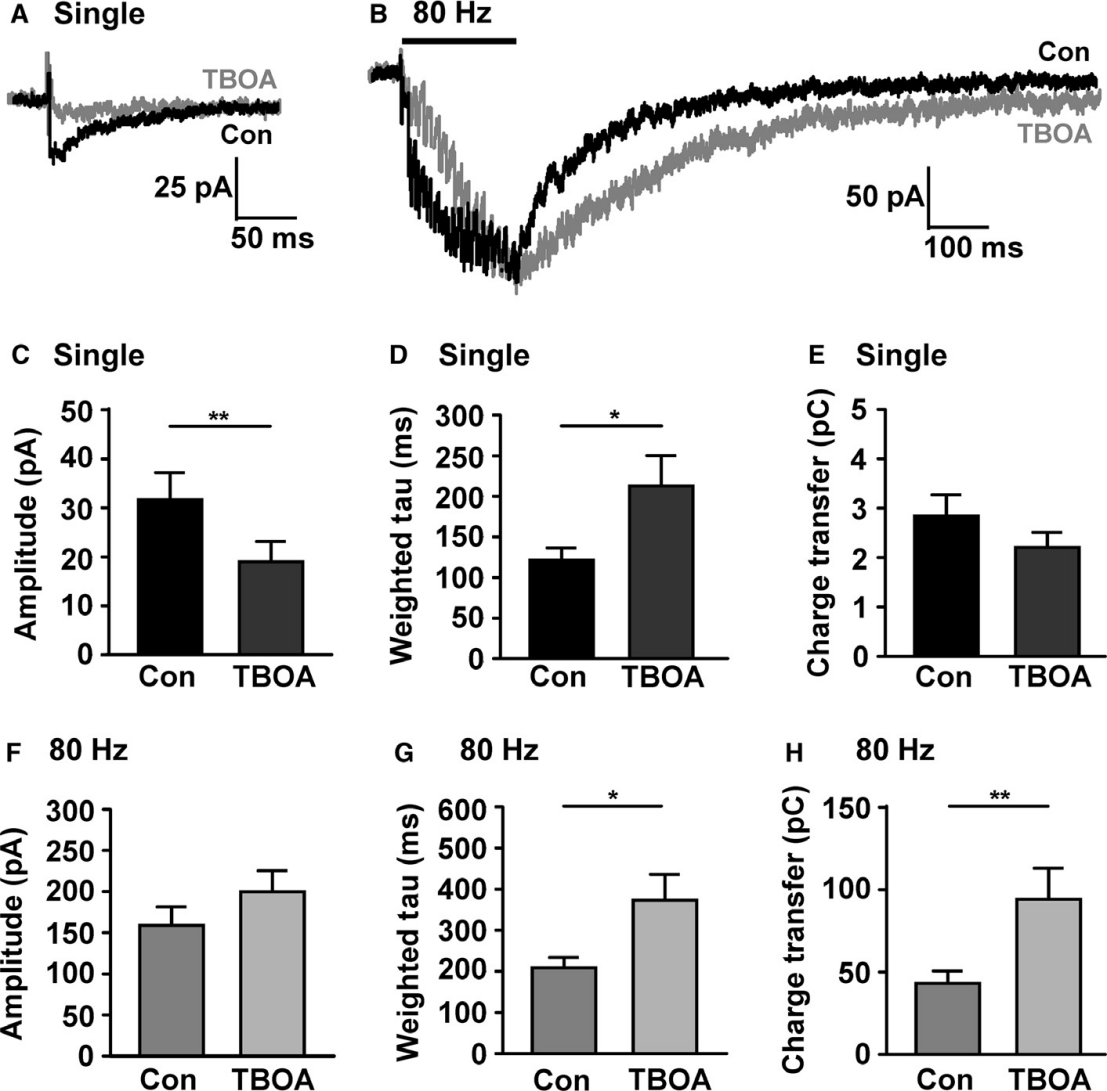
Inhibiting glutamate uptake increases the duration but not the amplitude of *N*‐methyl‐d‐aspartate glutamate receptor (NMDAR) responses in substantia nigra pars compacta (SNc) dopamine neurons. (A) Example recording from a SNc dopamine neuron of single NMDAR‐excitatory postsynaptic currents (EPSCs) in response to presynaptic stimulation (average of 10 responses delivered at 0.1 Hz) before (con) and after (TBOA) perfusion with TBOA (30 μm for 5 min). Current through synaptic NMDARs is reduced after TBOA. (B) Example recording from the same SNc dopamine neuron of a NMDAR‐EPSC in response to high‐frequency presynaptic stimulation (80 Hz for 200 ms; average of three responses) before (con) and after TBOA (30 μm for 5 min). The response is longer lasting but the peak amplitude does not change after TBOA. (C) Quantification of the peak amplitude in response to single stimuli (**P = 0.001). (D) Quantification of the decay time constant amplitude in response to single stimuli (*P = 0.03). (E) Quantification of the transfer of charge in response to single stimuli (*P *= 0.07). (F–H) As in (C–E), but for responses to 80‐Hz stimulation (*P *= 0.19 in F; *P = 0.02 in G; ** *P *= 0.01 in H; *n *= 9).

Next, the role of glutamate transporters in shaping the NMDAR‐EPSCs in SNc dopamine neurons in response to low‐ and high‐frequency stimulation was examined. The competitive antagonist of glutamate uptake, TBOA (30 μm; Herman & Jahr, 2007) was applied during 0.1‐Hz and 80‐Hz stimuli in the same dopamine neurons (Fig. 4; *n *= 9). In the presence of TBOA the amplitude of NMDAR‐EPSCs to 0.1‐Hz stimulation was significantly decreased (*P *= 0.001; paired *t*‐test; Fig. 4C), although the decay time was significantly increased (from 121.2 ± 15.6 ms to 212.9 ± 37.5 ms, *P *= 0.03, paired *t*‐test; Fig. 4D). There was no significant change in charge transfer (*P *= 0.07, paired *t*‐test; Fig. 4E). There was no significant effect of TBOA on the amplitude of NMDAR‐EPSCs to 80‐Hz stimulation (*P *= 0.19, paired *t*‐test; Fig. 4F), although the decay time was significantly increased (from 209.1 ± 25.1 ms to 373.7 ± 62.7 ms, *P *= 0.02, paired *t*‐test; Fig. 4G), as was the transfer of charge (*P *= 0.01, paired *t*‐test; Fig. 4H). The decrease in 0.1 Hz‐evoked NMDAR‐EPSC amplitude in TBOA suggested that other regulatory mechanisms may be engaged in the SNc when glutamate uptake is impaired.

**Figure 5:**
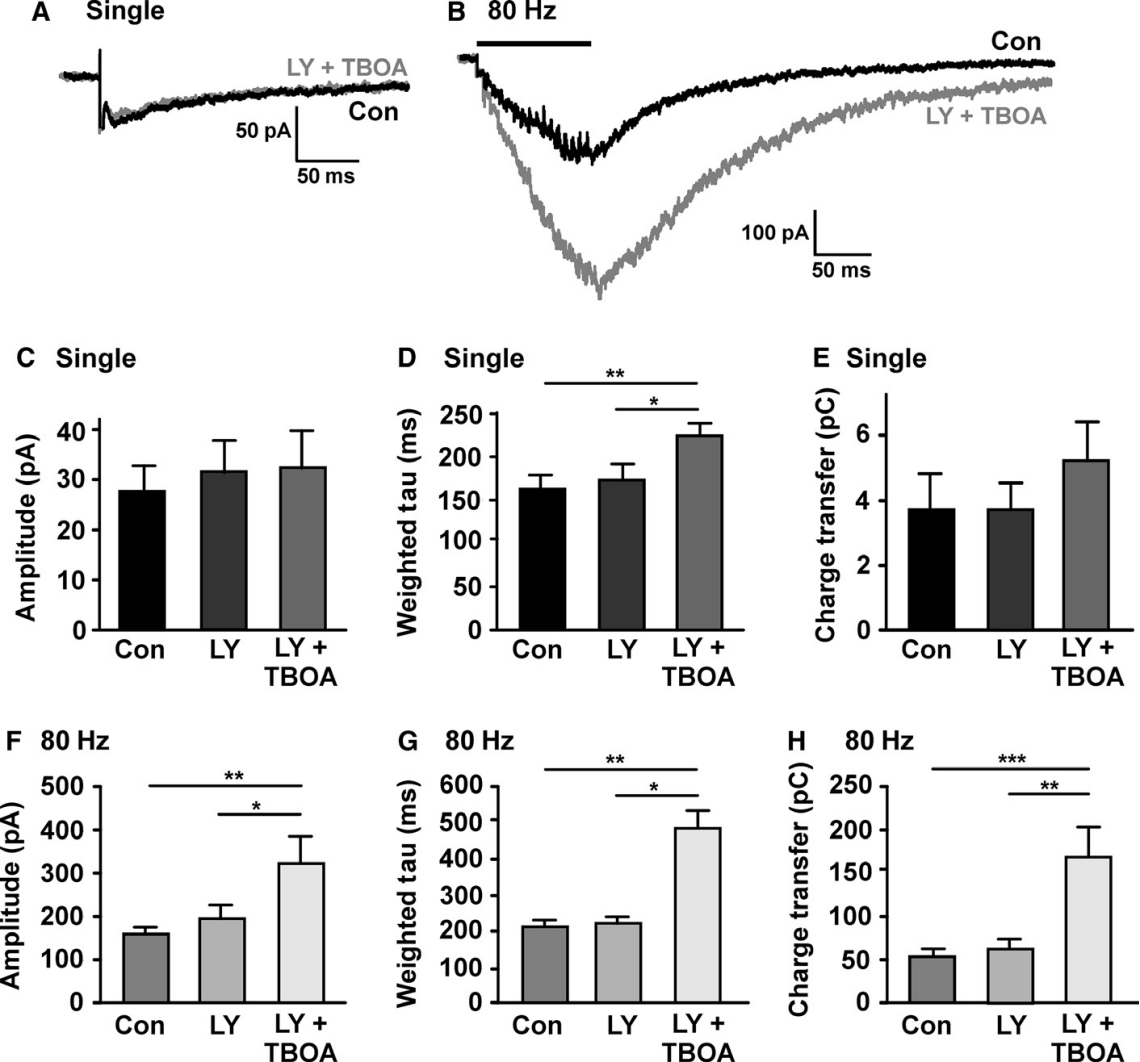
Regulation of *N*‐methyl‐d‐aspartate glutamate receptor (NMDAR) activity by the combined effect of glutamate transporters and Group II metabotropic glutamate receptors (mGluRs). (A) Example recording from a substantia nigra pars compacta (SNc) dopamine neuron of single NMDAR‐excitatory postsynaptic currents (EPSCs) in response to presynaptic stimulation (average of 10 responses delivered at 0.1 Hz) before (con) and after (LY + TBOA) perfusion with LY 341495 (200 nm for 5–10 min) and TBOA (30 μm for 5 min). Current through synaptic NMDARs is no longer reduced by TBOA in the presence of LY 341495. (B) Example recording from the same SNc dopamine neuron of a NMDAR‐EPSC in response to high‐frequency presynaptic stimulation (80 Hz for 200 ms; average of three responses) before (con) and after (LY + TBOA) perfusion with LY 341495 (200 nm for 5–10 min) and TBOA (30 μm for 5 min). The response is longer lasting and the peak amplitude is larger in TBOA plus LY 341495. (C) Quantification of the peak amplitude in response to single stimuli; there was no overall effect of LY or LY + TBOA (*P *= 0.71). (D) Quantification of the decay time constant in response to single stimuli showing a significant effect of TBOA plus LY 341495 (*P < 0.05; **P < 0.01). (E) Quantification of the transfer of charge in response to single stimuli; there was no overall effect of LY or LY + TBOA (*P *= 0.07). (F–H) As in (C–E), but for responses to 80‐Hz stimulation (*P < 0.05 and **P < 0.01 in F and G; **P < 0.01 and ***P < 0.001 in H; *n *= 9–10).

Presynaptic mGluRs are found on the glutamatergic terminals of subthalamic afferents to SNc, where they can mediate presynaptic inhibition of glutamate release (Bonci *et al*., 1997; Valenti *et al*., 2005; Wang *et al*., 2005). It was hypothesized that, in the experiments in Fig. 4, TBOA might have caused an accumulation of either evoked or tonic glutamate release to a sufficient concentration to recruit presynaptic mGluRs, inhibit glutamate release and cause the effect seen in Fig. 4A. To determine whether mGluRs were recruited by glutamate released during 0.1‐Hz and 80‐Hz stimulation, the Group II antagonist LY 341495 (200 nm; Wang *et al*., 2005) was applied; then TBOA was applied along with the LY 341495 (Fig. 5). Using repeated‐measures anova (or the non‐parametric Friedman test) to compare responses to single stimuli, there was no significant overall effect of LY 341495 plus TBOA on amplitude (Fig. 5C; repeated‐measures Friedman test, *F *= 0.8, *P *= 0.7). There was an overall significant effect on the decay time constant (Fig. 5D; repeated‐measures anova,* F *= 8.4, *P *= 0.003, total df* *= 26), although LY 341495 alone had no significant effect compared with control (Tukey *post hoc* test, *P *> 0.05). There was no overall effect on charge transfer (Fig. 5E; repeated‐measures anova,* F *= 3.1, *P *= 0.007 total df* *= 29). Comparing responses to 80‐Hz stimuli, there were overall significant effects on amplitude (Fig. 5F; repeated‐measures anova,* F *= 6.6, *P *= 0.007, total df* *= 29), decay time constant (Fig. 5G; Friedman test, *F *= 14, *P *= 0.0002) and charge transfer (Fig. 5H; repeated‐measures anova,* F *= 12.7, *P *= 0.0004, total df* *= 29). However, LY 341495 alone had no significant effect compared with control on amplitude, decay time or charge transfer (*P *> 0.05 in all *post hoc* tests). This suggests that under control conditions, glutamate concentration is insufficient to activate presynaptic Group II mGluRs and cause inhibition of glutamate release.

When TBOA was added to the LY 341495 perfusate (in the same neurons), no decrease in the amplitude of NMDAR‐EPSCs in response to 0.1‐Hz stimulation was seen (Fig. 5C); the decay time constant of NMDAR‐EPSCs in response to single stimuli was significantly increased (*P *< 0.01, Tukey *post hoc* test; Fig. 5D). In addition, the 80 Hz‐evoked NMDAR‐EPSC amplitude (−317.5 ±68.4 pA; Fig. 5F), decay time constant (481.0 ± 51.0 ms; Fig. 5G) and charge transfer (166.3 ± 34.4 pC; Fig. 5H) were all significantly larger in LY plus TBOA compared with control (*P *< 0.01, Tukey *post hoc* test; *P *< 0.01, Dunn's *post hoc* test; *P *< 0.001, Tukey *post hoc* test, respectively). These data indicate that Group II mGluRs are activated when glutamate transporters are compromised during high‐frequency stimulation, and that this can limit glutamate release, potentially helping to minimize spill‐over to extrasynaptic NMDARs.

**Figure 6:**
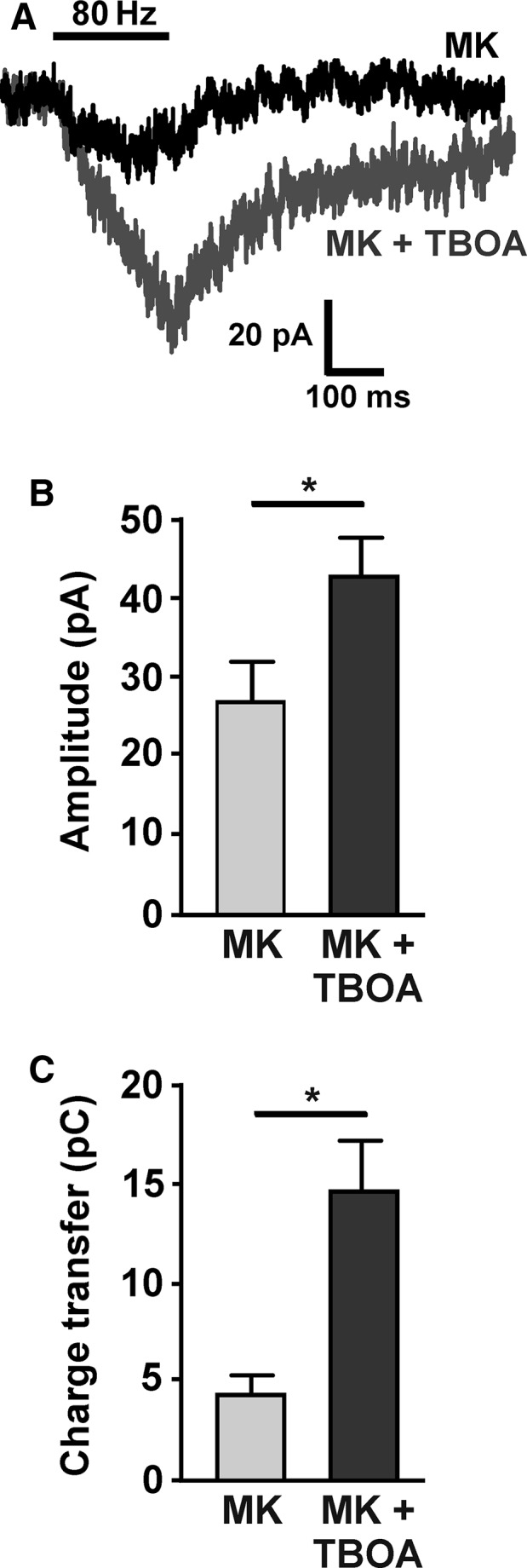
Recruitment of additional extrasynaptic *N*‐methyl‐d‐aspartate glutamate receptors (NMDARs) when glutamate transporters and Group II metabotropic glutamate receptors (mGluRs) are blocked. (A) Example recording from a substantia nigra pars compacta (SNc) dopamine neuron of a NMDAR‐excitatory postsynaptic current (EPSC) in response to high‐frequency presynaptic stimulation (80 Hz for 200 ms; average of three responses) after synaptic block using MK‐801 (MK; as in Fig. 2, but with 200 nm LY 341495) and subsequent perfusion with TBOA (30 μm for 5 min). The combination of LY 341495 plus TBOA significantly increased (B) the amplitude (*P = 0.03) and (C) the charge transfer (*P = 0.01) of the NMDAR response evoked by 80‐Hz stimuli after MK‐801 block of synaptic NMDARs (*n *= 7).

It was next determined whether the pool of extrasynaptic NMDARs remaining after MK‐801 block (Fig. 2C) could be enlarged by blocking glutamate transporters. The amplitude and charge transfer of 80 Hz‐evoked NMDAR‐EPSCs following MK‐801 block were significantly increased by subsequent application of TBOA (with LY 341495 applied throughout the experiment; Fig. 6; *n *= 7; *P *= 0.03 and *P *= 0.01, respectively, paired *t*‐tests). This suggests that additional extrasynaptic NMDARs can be recruited by 80‐Hz stimulation when mGluRs and/or glutamate transporters are compromised. These data confirm that under control conditions 80‐Hz stimulation does not recruit the total available pool of extrasynaptic NMDARs.

### Glutamate transporters regulate tonic NMDAR activity

**Figure 7:**
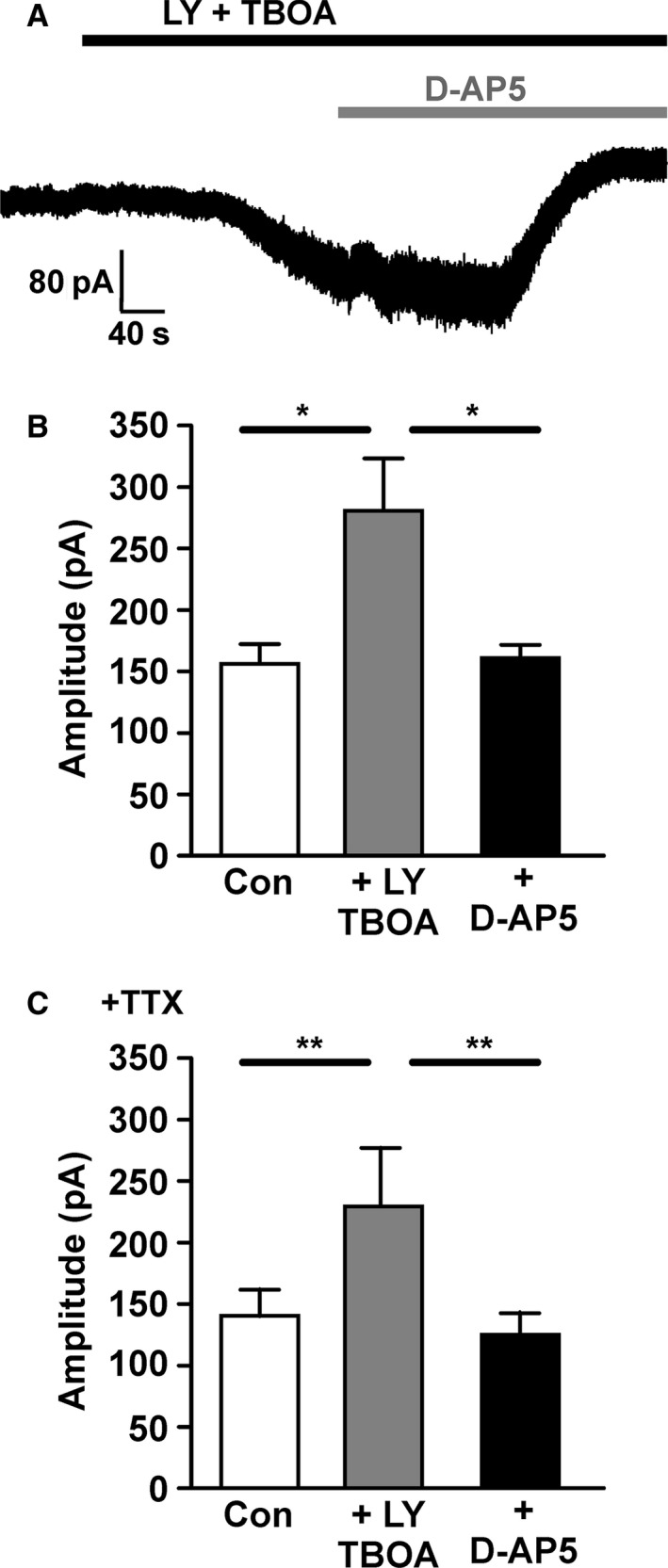
Glutamate transporters and group II metabotropic glutamate receptors (mGluRs) also regulate ambient glutamate and tonic activation of *N*‐methyl‐d‐aspartate glutamate receptors (NMDARs). (A) Example recording from a substantia nigra pars compacta (SNc) dopamine neuron of holding current at −50 mV (in control conditions: 0.1 mm Mg^2+^, 50 μm picrotoxin, 10 μm glycine and 10 μm DNQX) before and during perfusion of LY 341495 (200 nm) plus TBOA (30 μm) followed by D‐AP5 (50 μm). (B) Quantification of holding current at −50 mV in control conditions, in the presence of LY plus TBOA, and in LY, TBOA and D‐AP5. LY plus TBOA caused a significant inward current, and D‐AP5 caused a significant reduction of LY/TBOA‐induced inward current (*n *= 10; *P < 0.05). (C) Quantification of holding current as in (B), but in the presence of TTX (100 nm;* n *= 9; **P < 0.01).

The small but significant D‐AP5‐sensitive current shown in Fig. 3 suggests that tonic glutamate release, possibly non‐vesicular in origin, occurs in the SN. The decrease in single NMDAR‐EPSC amplitude after TBOA (Fig. 4C) suggested that TBOA might cause an accumulation of extracellular glutamate that in turn causes presynaptic inhibition of glutamate release. Therefore, next it was examined whether tonic NMDAR activity is regulated by glutamate transporters. Using repeated‐measures anova (or the non‐parametric Friedman test) to determine the effects of TBOA (plus LY 341495) followed by D‐AP5 on holding current, an overall significant effect was observed (Fig. 7B; anova,* F *= 6.5, *P *= 0.008, total df* *= 29), with a significant inward current of −124.6 ±49.4 pA (*P *< 0.05) that was significantly reduced by D‐AP5 to −4.8 ± 14.8 pA (*P *< 0.05, Tukey *post hoc* test). In the presence of TTX (Fig. 7C), there was also an overall significant effect (Friedman test, *F *= 13.6, *P *= 0.0003). TBOA (plus LY 341495) evoked an inward current of 89.2 ± 42.9 pA (*P *< 0.05) that was reduced by D‐AP5 to 15.9 ± 14.5 pA (*P *< 0.05, Dunn's *post hoc* test). Overall, the data suggest that LY–TBOA caused an inward current that is not significantly different in amplitude when action potential‐dependent glutamate release is blocked with TTX, and that this current was largely mediated by NMDARs.

## Discussion

The contribution of NMDARs distal to synapses in SNc dopamine neurons to responses evoked by synaptic glutamate release during low‐ (0.1 Hz) and high‐ (80 Hz) frequency presynaptic stimulation has been investigated. Recruitment of putative extrasynaptic NMDARs by 80‐Hz stimulation appears to be regulated by binding or removal of extracellular glutamate via transporters, and by mGluRs that limit presynaptic glutamate release. These mechanisms may be essential for limiting glutamate diffusion away from synaptic sites in SNc dopamine neurons and thus shaping the NMDAR‐mediated EPSC.

### High‐frequency stimulation recruits at least a small population of extrasynaptic NMDARs

It has previously been shown that 80‐Hz stimulation of excitatory inputs to rat SNc dopamine neurons evokes NMDAR‐EPSCs that are more susceptible to memantine block than single NMDAR‐EPSCs (Wild *et al*., 2013). In this study, in mouse SNc dopamine neurons, it was found that NMDAR‐EPSCs in response to 80‐Hz stimulation were significantly larger in amplitude and longer in duration than responses to single stimuli, allowing significantly more charge to be transferred. One possible explanation for this is that during 80‐Hz stimulation glutamate diffuses beyond the synapse and activates NMDARs that are distal to the synapse, so‐called extrasynaptic NMDARs, effectively increasing the number of NMDARs contributing to the NMDAR‐EPSC. By using MK‐801 to block activated synaptic NMDARs (during 0.1‐Hz stimulation) to determine extrasynaptic NMDAR activity during 80‐Hz stimulation, it was found that 8–16% of the response to 80‐Hz stimulation remained after MK‐801 block and thus appears to be extrasynaptic. Memantine inhibited the 80 Hz‐evoked NMDAR current remaining after MK‐801 block, and the percent inhibition (53%) was within the range of memantine inhibition of the full response (synaptic plus extrasynaptic) to 80‐Hz stimulation (39%) and of whole cell NMDAR responses (48% and 66% when using bath or picospritzer application of NMDA, respectively) in rat SNc dopamine neurons (Wild *et al*., 2013). This supports the idea that extrasynaptic NMDARs are susceptible to memantine inhibition (Xia *et al*., 2010; Wu & Johnson, 2015), although memantine inhibition of the 80 Hz‐evoked synaptic NMDAR population cannot be ruled out.

It is possible that a diluted concentration of glutamate reaches extrasynaptic NMDARs, allowing them to contribute only a small proportion of the response to 80‐Hz stimulation. It is also possible that, during 0.1‐Hz stimulation in the presence of MK‐801, some extrasynaptic NMDARs were activated and therefore blocked. However, the possibility that ambient glutamate might cause tonic NMDAR activity was also considered, enabling widespread MK‐801 block, and causing the underestimation of the percentage of extrasynaptic NMDARs. In agreement with this, a significant tonic D‐AP5‐sensitive NMDAR current was measured, and significant MK‐801 block was subsequently observed even in the absence of synaptic stimulation. Therefore, it was concluded that 80 Hz synaptic stimulation releases sufficient glutamate to diffuse to and activate NMDARs distal to the synapse, that this population of so‐called extrasynaptic NMDARs forms at least 8–16% of those underlying the 80‐Hz‐evoked NMDAR‐EPSC, and that it can be inhibited by memantine. However, it seems likely that the actual proportion of extrasynaptic NMDARs activated by 80‐Hz stimulation is higher than 8–16%. It would be recommended to test whether there is tonic NMDAR activity before using MK‐801 to calculate the proportion of synaptic and extrasynaptic NMDARs activated by glutamate release.

Compared with the understanding of the organization and function of NMDARs in and near synapses in spiny dendrites (Lau & Zukin, 2007), much less is known about aspiny neurons. SNc dopamine neurons have aspiny dendrites (although they occasionally have sparse dendritic ‘appendages’; Tepper *et al*., 1987). Electron micrographic studies indicate that NMDA [and α‐amino‐3‐hydroxy‐5‐methyl‐4‐isoxazolepropionic acid (AMPA)] receptor subunits are found away from asymmetric synapses (Paquet *et al*., 1997; Chatha *et al*., 2000); these may represent extrasynaptic receptors. While AMPAR‐EPSCs have not been studied, the current data do provide the first functional evidence suggesting that extrasynaptic NMDARs contribute to the shape of the NMDAR‐EPSC in SNc dopamine neurons during high‐frequency stimulation. A recent study has visualized endogenously expressed fluorescently labelled postsynaptic density (PSD)‐95 protein in aspiny dopam‐ine neurons (Fortin *et al*., 2014), and the synapses appear to be 1–2 μm apart; using this tool, future work will hopefully elucidate the expression of glutamate receptor proteins at different distances from the PSD.

### Mechanisms regulating glutamate spill‐over

In models of neurotransmitter diffusion, glutamate spill‐over from the synaptic cleft can occur within 50 μs after release if uptake mechanisms are absent (Clements, 1996). TBOA was used to explore the importance of glutamate transporters in shaping NMDAR‐EPSCs in SNc dopamine neurons. TBOA alone had little effect on the amplitude of NMDAR responses to high‐frequency stimulation, and caused a significant decrease in the response to single stimuli. TBOA appeared to be binding to glutamate transporters, because the NMDAR response decay was significantly slower. It was hypothesized that by increasing the duration and possibly the diffusion distance of extracellular glutamate, TBOA might enable glutamate to bind to presynaptic receptors that inhibit glutamate release, and that this might offset the potential activation of extrasynaptic NMDARs. mGluRs are present on the terminals of subthalamic inputs to dopamine neurons and can modulate glutamate release (Bonci *et al*., 1997; Valenti *et al*., 2005; Wang *et al*., 2005). It was found that the Group II mGluR antagonist LY 341495 alone had no effect on NMDAR‐EPSCs. Interestingly, Wang *et al*. (2005) found that LY 341495 increases the amplitude of AMPA‐EPSCs in response to single stimuli in dopamine neurons, and increases the frequency of AMPA receptor‐mediated miniature EPSCs. One possibility is that in the current experiments glutamate released by single or 80‐Hz stimuli did not reach presynaptic Group II mGluRs, for example if they are located distal to the active zone. In support of this, TBOA applied with LY 341495 no longer reduced single NMDAR‐EPSCs and now caused a significant increase in 80 Hz‐evoked NMDAR‐EPSCs. TBOA when applied with LY 341495 did not potentiate single NMDAR‐EPSCs; this supports the idea that glutamate transporters have little influence on the decay of single NMDAR‐EPSCs (Clements, 1996; Barbour, 2001). Overall the current data suggest that in SNc dopamine neurons, compromising glutamate transporters may enable ambient glutamate to increase, activating presynaptic mGluRs and decreasing glutamate release. This may be an additional regulatory mechanism, alongside transporter activity, to ensure glutamate levels do not increase further when uptake is compromised, preventing released glutamate from accessing extrasynaptic NMDARs.

A combined mechanism of transporter and mGluR control of EPSC shape has been described in developing retinogeniculate synapses (Hauser *et al*., 2013). This mechanism may be important in SNc dopamine neurons for keeping synaptic responses to action potential‐dependent glutamate release relatively small, fast and specific. It may ordinarily limit the activation of extrasynaptic NMDARs, preventing them from contributing to synaptic signalling, and ensuring that synaptic NMDARs are the main triggers of burst firing (Blythe *et al*., 2007) and long‐term potentiation (Bonci & Malenka, 1999; Harnett *et al*., 2009) so that only salient synaptic inputs influence dopaminergic output to the striatum. On the other hand, although spill‐over can potentially reduce the specificity of synaptic signalling (Asztely *et al*., 1997; Barbour, 2001; Diamond, 2002; Herman & Jahr, 2007; Scimemi *et al*., 2009), it may allow for inter‐synaptic cooperativity (Arnth‐Jensen *et al*., 2002; Diamond, 2002). Therefore, a degree of glutamate spill‐over could be important in the substantia nigra, for example if co‐ordinated activation of populations of dopamine neurons is required, and this could be controlled via modulation of glutamate transporter and/or mGluR activity.

The mechanisms regulating glutamate spill‐over in the SNc may also limit activation of extrasynaptic NMDARs that can couple to cell death signalling pathways and promote excitotoxicity (Hardingham & Bading, 2010; Wyllie *et al*., 2013), particularly under pathological conditions that challenge synapse function; for example, when excitatory drive to dopamine neurons from the subthalamic input is increased or when mitochondria function is compromised, which reduces ATP levels and disrupts the ionic gradients on which glutamate transporters depend. Both of these situations can occur in human patients with PD and in animal models of PD (Magnin *et al*., 2000; Obeso *et al*., 2010; Piallat *et al*., 2011). Inhibition of glutamate transporters in SNc induces PD‐like signs in rats, in part due to NMDAR‐mediated excitotoxicity (Assous *et al*., 2014). More generally, activators of glutamate transporters have therapeutic potential, showing promise in animal models of neurodegeneration (Jensen *et al*., 2015).

### Sources of ambient glutamate

The current data support the presence of action potential‐independent ambient glutamate causing tonic NMDAR activity in SNc dopamine neurons, and this appears to be regulated by glutamate transporters. Ambient levels of extracellular glutamate and tonic NMDAR activity in the hippocampus (Sah *et al*., 1989) are increased by inhibitors of glutamate uptake, but blocking sodium channels or vesicular release has no effect (Herman & Jahr, 2007; Le Meur *et al*., 2007), supporting the idea that ambient glutamate does not originate from action potential‐dependent release from neurons, but is non‐neuronal in origin, possibly released from glia (Parpura *et al*., 1994; Bezzi *et al*., 1998; Angulo *et al*., 2004; Fellin *et al*., 2004).

In conclusion, mGluR signalling and glutamate transporters in the SNc may restrict the diffusion of glutamate to extrasynaptic NMDARs and limit excitotoxic signalling in dopamine neurons. The failure of these mechanisms could contribute to the declining health of dopamine neurons during pathological conditions. An intriguing question for future research will be whether the activity of extrasynaptic NMDARs is increased in animal models of PD.
